# Retrospective *post-hoc* subgroup analysis of adjunctive non-invasive vagus nerve stimulation in chronic mTBI with comorbid PTSD

**DOI:** 10.3389/fnins.2026.1808542

**Published:** 2026-04-13

**Authors:** Michael Ament, Emily Leonard, Peter S. Staats, Norianne T. Ingram

**Affiliations:** 1 Cherry Creek Neurology, Denver, CO, United States; 2 Vagus Nerve Society, Atlantic Beach, FL, United States; 3 electroCore, Inc., Rockaway, NJ, United States

**Keywords:** concussion, neuroinflammation, neuromodulation, non-invasive vagus nerve stimulation, nVNS, persistent symptoms, PTSD, traumatic brain injury

## Abstract

**Introduction:**

Persistent symptoms following mild traumatic brain injury (mTBI) remain a major clinical challenge. Patients with comorbid post-traumatic stress disorder (mTBI + PTSD) typically exhibit greater symptom burden and poorer outcome. Overlapping pathology—dysfunction in central autonomic, limbic, and cognitive networks—suggests a potential role for neuromodulatory interventions. Non-invasive vagus nerve stimulation (nVNS) has shown promise as a treatment for both neurotraumatic and psychiatric conditions, but its real-world impact in patients with chronic mTBI and comorbid PTSD has not been well characterized.

**Methods:**

This study represents a post-hoc subgroup analysis of a previously published retrospective observational cohort in adults with chronic mTBI symptoms treated with adjunctive nVNS. Symptom severity was assessed using the Neurobehavioral Symptom Inventory (NSI) at baseline and after 3–4 months of treatment. A post-hoc PTSD-enriched subgroup was identified using the PTSD Checklist for DSM-5 (PCL-5; score ≥31). Changes in total NSI scores, symptom domains, and item-level responses were evaluated for improvement. Multivariate analyses were used to characterize baseline concussion symptom profiles with (PTSD+) and without PTSD.

**Results:**

Among PTSD+ patients, adjunctive nVNS was associated with a significant reduction in overall symptom burden, with mean total NSI scores decreasing from 2.50 ± 0.60 at baseline to 2.03 ± 0.46 at follow-up (FDR-corrected, *q* < 0.05). Improvements were most pronounced in affective (−0.58, *q* = 0.010) and cognitive (−0.64, *q* = 0.015) symptom domains, with additional reductions in somatic (−0.34, *q* = 0.040) and vestibular (−0.58, *q* = 0.050) symptoms. Forty percent of PTSD+ patients achieved a ≥30% reduction in total symptom burden, and 29% demonstrated potentially clinically meaningful improvement across 50% or more of tracked symptoms. Multivariate analyses showed that PTSD+ patients clustered within a high–symptom-burden phenotype that exhibited comparable absolute symptom reductions.

**Conclusion:**

Adjunctive nVNS was well tolerated and associated with symptom improvement in patients with mTBI + PTSD, a population often considered refractory to treatment. These findings support the feasibility of vagal neuromodulation and highlight a high-symptom-burden subgroup with distinct symptom patterns that may retain treatment responsiveness, warranting further prospective evaluation.

## 1 Introduction

Traumatic brain injury (TBI) is a prevalent neurological condition associated with significant long-term morbidity. While most cases are classified as mild (mTBI), a meaningful subset of patients develop persistent symptoms affecting cognitive, emotional, and physical domains (Polinder et al., 2018; Voormolen et al., 2018). Longitudinal studies suggest that 10–25% of individuals with mTBI experience symptoms beyond the acute recovery period (McInnes et al., 2017), contributing to increased disability, reduced quality of life, and an estimated $76.5 billion in annual economic burden in the United States alone (Finkelstein et al., 2006). An increasingly recognized dimension of this burden is the high rate of psychiatric comorbidities—most notably post-traumatic stress disorder (PTSD)—which is observed in up to 35% of individuals with TBI and in as many as 65% of military service members with combat-related mTBI (Ruff et al., 2012; Chen and Zhao, 2019). Comorbidity is associated with worse functional outcomes than either condition alone, and substantial symptom overlap (e.g., sleep disturbance, irritability, attentional deficits, and emotional dysregulation) complicates both diagnosis and treatment planning (Amen et al., 2015; Zhang et al., 2021).

Treatment of persistent mTBI symptoms remains a clinical challenge, particularly when complicated by comorbid psychiatric conditions such as PTSD. Standard care is largely supportive, with limited efficacy in chronic presentations (U.S. Department of Veteran Affairs, 2021, 2023). Pharmacologic interventions are often ineffective or poorly tolerated, while behavioral therapies may be insufficient on their own. This therapeutic gap is especially pronounced in patients with dual diagnoses, where overlapping neurobiological and psychological mechanisms contribute to a more refractory clinical course (Dieter and Engel, 2019; Peitz et al., 2021). In response, there is growing interest in targeted interventions that modulate shared pathophysiological circuits.

Comorbid mTBI and PTSD share overlapping pathophysiological features, including autonomic dysregulation, limbic hyperreactivity, neuroinflammatory priming, and Hypothalamic–Pituitary–Adrenal axis dysfunction which contribute to persistent emotional and cognitive symptoms (Dieter and Engel, 2019; Jurick et al., 2021; Gillam et al., 2023; Daugherty et al., 2024). The vagus nerve may serve as a key interface among these disrupted systems. Neural circuits involved in stress and emotion regulation, such as the medial prefrontal cortex, amygdala, and hippocampus, are vulnerable in both conditions and modulated by vagal input (Bremner, 2007; Dieter and Engel, 2019; Smith et al., 2023). These shared mechanisms provide a rationale for neuromodulatory interventions targeting vagal pathways.

gammaCore™, a handheld, non-invasive vagus nerve stimulator with a patented frequency and waveform, engages this circuitry through afferent vagal activation. Preclinical models demonstrate that nVNS reduces neuroinflammation, preserves blood–brain barrier integrity, and modulates limbic and autonomic function in both TBI and PTSD (Yang et al., 2018; McIntrye, 2019; Morais et al., 2024). Clinically, gammaCore has shown efficacy in related disorders such as migraine and has received FDA Breakthrough Device Designation for PTSD (Silberstein et al., 2016; Bremner et al., 2021; Najib et al., 2022). Across multiple clinical trials, gammaCore has been shown to have a well-established safety profile (Gaul et al., 2016; Arsava et al., 2022) including in adolescent patients (Grazzi et al., 2017). While no serious adverse events have been reported, minor transient and time-limited side effects may occur. The most common of these include minor skin irritation at the application site and a “lip pull” ipsilateral to the side of stimulation may occur in ~85% of users. The lip pull resolves at the offset of stimulation. Its favorable safety profile and mechanistic plausibility make gammaCore a promising candidate for intervention in this population.

In a prior real-world observational study, adjunctive nVNS was well tolerated and associated with progressive symptom reduction in patients with chronic mTBI (Ament et al., 2025). Although the cohort was heterogeneous and many participants reported symptoms overlapping with PTSD, comorbidity was not a primary focus of that analysis. The present work represents a secondary post-hoc subgroup analysis of this previously published uncontrolled observational cohort and specifically evaluates patients with chronic mTBI who exhibit clinical features consistent with PTSD. Using patient-reported trajectories and Neurobehavioral Symptom Inventory (NSI) subscales (King et al., 2012; Soble et al., 2014), we examine the real-world use of nVNS on persistent symptoms that stem from a complex interaction between neurological injury and psychological trauma.

## 2 Methods

### 2.1 Study design and participants

This analysis represents a *post-hoc* subgroup evaluation of data originally reported in Ament et al. (2025), a previously published uncontrolled observational cohort. The source population included adults with chronic mild traumatic brain injury (mTBI) treated at a concussion-focused neurology practice (Cherry Creek Neurology, Denver, CO, United States) between October 2020 and September 2024. The practice primarily manages patients in the context of personal injury evaluation and treatment.

Adjunctive non-invasive vagus nerve stimulation (nVNS; gammaCore™, electroCore, Inc.) was offered as part of routine clinical care to patients who demonstrated more prominent affective symptom burden (e.g., depression and/or anxiety) in the setting of persistent post-concussive symptoms. Thus, the present sample reflects a clinically indicated, non-consecutive subgroup rather than an unselected chronic mTBI population.

nVNS was administered in addition to standard of care (SoC), which consisted of individualized multimodal management including pharmacologic treatment, behavioral therapy, vestibular/physical therapy, and other symptom-targeted interventions as clinically indicated (see Supplementary Table 2).

All patients provided written informed consent for research use of de-identified clinical and survey data. The study was conducted under an Institutional Review Board–approved protocol (IRB# 202301118, University of Florida).

Participants were adults with symptoms persisting ≥3 months following mTBI. Patients were not considered for nVNS therapy if they had contraindications including implanted pacemaker or defibrillator, other implanted medical devices (excluding orthopedic hardware), prior cervical spinal fusion or surgery at or above the C4 vertebra, prior anterior neck surgery (e.g., radical neck dissection or carotid surgery), or current pregnancy. Of 175 patients who received nVNS during the study period, 102 had complete NSI data and were included in this post-hoc analysis.

### 2.2 PTSD subgroup

A subset of patients completed the PTSD Checklist for Diagnostic and Statistical Manual of Mental Disorders (DSM)-5 (PCL-5; *n* = 54) (Weathers et al., 2013). PCL-5 screening was administered based on clinical suspicion of affective distress rather than systematically to all participants, and therefore represents a selectively assessed subgroup. Of those 54 patients, only 42 had complete NSI data at both intake and follow up and 12 more patients were excluded from final analyses. Participants with PCL-5 ≥ 31 were classified as having comorbid PTSD (PTSD^+^; *n* = 35; Supplementary Table 1), consistent with accepted criteria; those scoring <31 comprised the mTBI-only group (PTSD^−^; *n* = 7). Analyses were performed within and between subgroups (PTSD^+^ vs. PTSD^−^), with additional structural and sensitivity analyses incorporating participants without PCL-5 data (PTSD^N/A^; *n* = 111).

Primary subgroup comparisons involving PTSD status were restricted to participants with available PCL-5 data (PTSD^+^ vs. PTSD^−^). Participants without PCL-5 data (PTSD^N/A^) were included in principal component and sensitivity analyses to evaluate overall symptom structure and robustness of findings but were not used to infer PTSD-related treatment effects.

### 2.3 Intervention

The nVNS protocol was identical to that reported in Ament et al. (2025). Patients self-administered two consecutive 2-min stimulations to the cervical vagus nerve twice daily, with optional additional treatments for symptom flares. nVNS was used adjunctively with individualized SoC management.

### 2.4 Outcome measures

Primary outcome was change in total NSI score between baseline and 3–4-month follow-up. Secondary outcomes included NSI domain-level and item-level changes. NSI domains (vestibular, somatic, cognitive, and affective; see Supplementary Table 3) were defined *a priori* based on standard NSI item groupings as reflected in Table 1. Domain composition was not modified for the purposes of this post-hoc analysis.

**Table 1:** NSI symptom domains and average severity at baseline and after treatment with adjunctive nVNS.

NSI domains	Baseline NSI mean	Baseline NSI STD	Follow-Up NSI mean	Follow-Up NSI STD	*p*-value	FDR-adjusted *p*-value
Dizziness	2.46	0.98	2.00	1.11	0.060	0.083
Loss of balance	**2.40**	**1.14**	**1.71**	**0.93**	**0.003**	**0.017**
Poor coordination	**2.46**	**0.92**	**1.86**	**1.06**	**0.002**	**0.017**
Post-traumatic headaches	**3.17**	**0.79**	**2.46**	**1.07**	**0.004**	**0.017**
Nausea	1.80	1.18	1.49	1.22	0.220	0.243
Vision problems	1.94	1.16	2.26	0.95	0.098	0.127
Light sensitivity	2.37	1.06	2.06	1.03	0.177	0.205
Difficulty hearing	1.51	1.15	1.37	0.97	0.554	0.554
Sensitivity to noise	**2.40**	**1.12**	**1.89**	**1.08**	**0.027**	**0.046**
Numbness/tingling	2.06	1.28	1.51	1.38	0.037	0.057
Altered taste/smell	1.06	1.20	0.77	1.09	0.149	0.182
Appetite	1.83	1.20	1.66	1.16	0.397	0.415
Poor concentration	**2.97**	**0.98**	**2.34**	**1.06**	**0.015**	**0.033**
Forgetfulness	**3.14**	**0.85**	**2.49**	**1.12**	**0.004**	**0.017**
Decision making	**2.69**	**0.83**	**2.06**	**1.03**	**0.017**	**0.034**
Slowed thinking	**2.97**	**0.79**	**2.34**	**1.06**	**0.008**	**0.026**
Fatigue	**3.23**	**0.84**	**2.60**	**1.01**	**0.006**	**0.023**
Falling asleep	**3.00**	**1.03**	**2.40**	**1.06**	**0.010**	**0.029**
Anxious/tense	**3.03**	**0.92**	**2.51**	**0.98**	**0.015**	**0.033**
Depressed sad	**2.63**	**1.21**	**2.11**	**1.18**	**0.022**	**0.041**
Irritability	2.83	1.01	2.31	1.18	0.040	0.058
Easily overwhelmed	**3.11**	**0.93**	**2.40**	**1.17**	**0.001**	**0.017**
Vestibular score	**7.31**	**2.72**	**5.97**	**2.83**	**0.0495**	**0.049**
Somatic score	**14.80**	**5.22**	**12.57**	**5.27**	**0.030**	**0.040**
Cognitive score	**11.77**	**2.76**	**9.17**	**4.00**	**0.0076**	**0.015**
Affective score	**17.83**	**4.21**	**13.80**	**6.39**	**0.0025**	**0.010**
Total score	**55.18**	**13.00**	**44.41**	**17.36**	**0.0034**	**–**

NSI symptom domains (rows 1–22) and the average PTSD^+^ patient scores (*n* = 35) with standard deviations. Wilcoxon signed-rank was used to compare means. Bolded rows show items that met significance after using FDR to correct for multiple comparisons (FDR-adjusted *p* < 0.05). Rows 23–26 show composite scores based on select symptoms listed above. Total score combines all 22 NSI symptoms. Standard deviation (STD).

Exploratory analyses in the mTBI + PTSD subgroup focused on cognitive and affective symptom trajectories.

### 2.5 Statistical analysis

Analyses were conducted using custom scripts written in MATLAB (MathWorks, Natick, MA). Continuous variables are reported as mean ± standard deviation unless otherwise specified. Two-tailed tests were applied, with statistical significance defined as *p* < 0.05. The false discovery rate (FDR; Benjamini–Hochberg, *q* < 0.05) was used to control for multiple comparisons where applicable.

Within-group changes in total NSI and domain scores were assessed using Wilcoxon signed-rank tests or analysis of variance (ANOVA), as appropriate. Pearson correlation coefficients were calculated to examine associations between baseline symptom severity and treatment-related change.

To account for potential regression-to-the-mean effects and baseline severity differences between subgroups, analysis of covariance (ANCOVA) models were performed. Follow-up NSI scores were modeled as a function of baseline NSI and group status:


$$\text{Follow}-\mathrm{up}\;\mathrm{NSI}=\beta_{0}+\beta_{1}(\text{Baseline}\;\mathrm{NSI})+\beta_{2}(\text{Group})$$

Baseline × Group interaction terms were included to assess homogeneity of regression slopes. Primary ANCOVA models compared mTBI-only and mTBI + PTSD subgroups. Sensitivity analyses incorporated a third group consisting of patients without available PCL-5 data to evaluate robustness of findings to incomplete PTSD ascertainment.

Domain-level ANCOVA models were conducted using the same framework to determine whether PTSD status was associated with differential follow-up severity within cognitive, affective, somatic, or vestibular symptom domains. This approach evaluates follow-up severity while statistically controlling for baseline differences, thereby accounting for baseline severity differences and potential regression-to-the-mean effects.

### 2.6 Principal component analysis

Principal component analysis (PCA) was performed on baseline NSI item scores to identify underlying symptom dimensions and assess whether the mTBI + PTSD subgroup exhibited distinct multivariate symptom profiles. Components were extracted using singular value decomposition after *z*-score normalization of variables. The first three principal components were retained for interpretation based on eigenvalues >1 and plot inspection. Component loadings were examined to characterize symptom clusters, and group separation was visualized in three-dimensional PC space.

Stability of the PCA solution was assessed using 2,000 bootstrap resamples with replacement. Component loadings were aligned to the reference solution to account for potential sign indeterminacy.

### 2.7 Safety assessments

No device-related adverse events were reported during the 3-month treatment period.

## 3 Results

### 3.1 Cohort composition and identification of the mTBI + PTSD subgroup

Data were analyzed from 175 patients with chronic mild traumatic brain injury as previously reported in Ament et al. (2025). Briefly, these patients received adjunctive non-invasive vagus nerve stimulation (nVNS) with personalized SoC at Cherry Creek Neurology between October 2020 and September 2024. Of these patients, forty-two participants completed the PTSD Checklist (PCL-5), and 35 (83%) scored ≥31 (average 50.86 ± 13.00), meeting criteria for probable post-traumatic stress disorder (PTSD). This high incidence of probable PTSD reflects the selective screening criteria for PCL-5 administration (i.e., suspected PTSD or severe affective symptoms).

The mTBI + PTSD subgroup demonstrated higher baseline NSI total scores (PTSD^+^; 2.50 ± 0.60; *n* = 35) compared with patients without PTSD (PTSD^−^; 2.09 ± 0.76; *n* = 7) or patients without PCL-5 data (PTSD^N/A^; 1.79 ± 0.55; *n* = 111), reflecting greater symptom burden at study entry. A one-way ANOVA revealed a significant group effect, *F*(2,121) = 14.90, *p* < 0.001. Tukey post-hoc comparisons indicated that PTSD^+^ patient baseline symptom severity was significantly higher than the baseline severity of PTSD^N/A^ patients (*p* < 0.001). No other pairwise comparisons were significant.

Attempts were made to track prior exposure and concurrent use of medications and other therapies are summarized in Supplementary Table 2. We did not see any effect of prior or current psychotherapy or psychiatric medications on baseline or follow up affective scores (data not shown). A similar analysis for vestibular medications showed that individuals who received vestibular therapy (*n* = 15) tended to present with more severe vestibular domain scores (8.27 ± 1.87 vs. 6.60 ± 3.07).

### 3.2 Symptom change following adjunctive nVNS in patients with mTBI + PTSD

**Figure 1:**
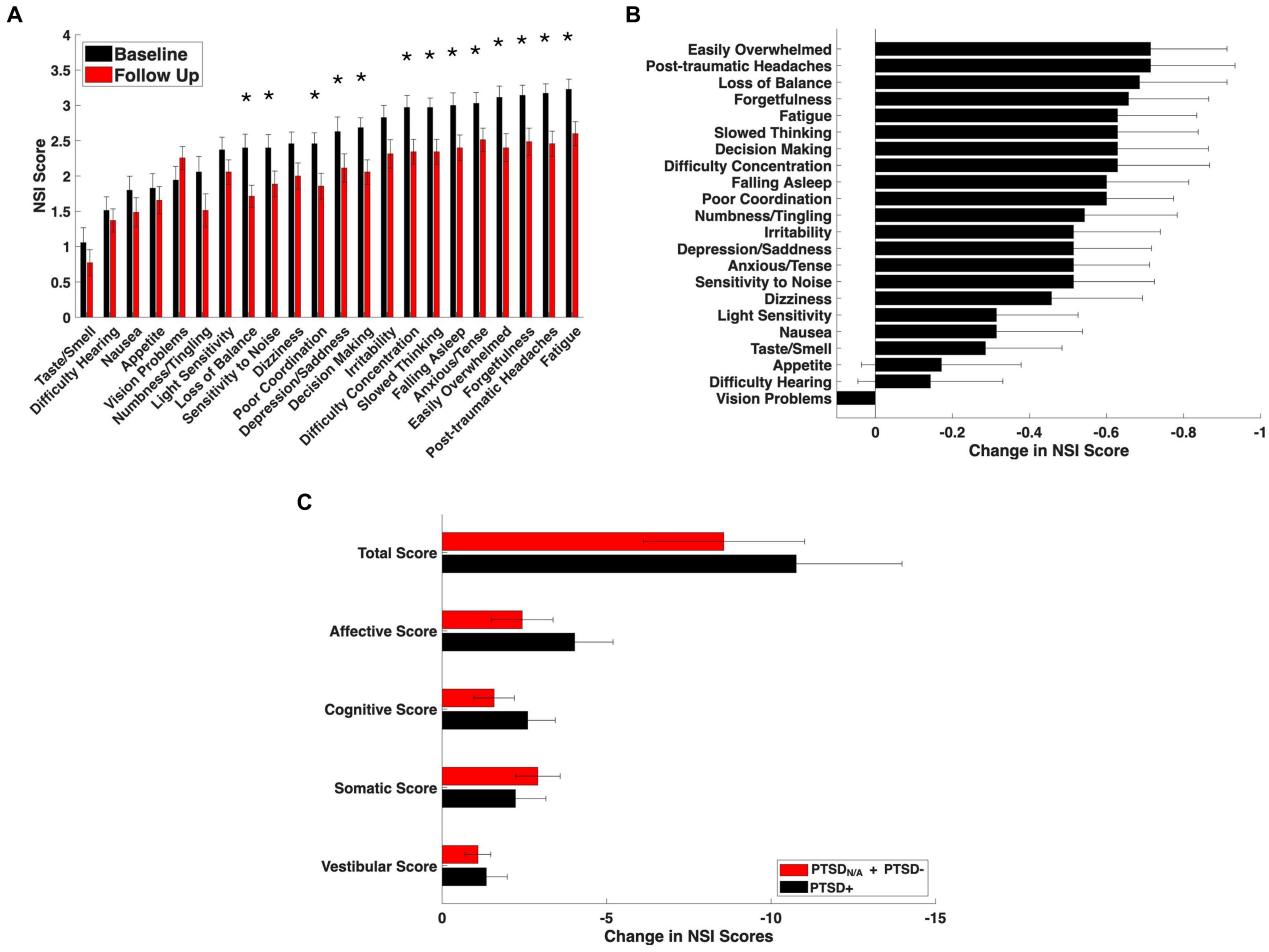
Symptom burden at baseline and after adjunctive nVNS treatment in patients with mTBI + PTSD. **(A)** NSI scores across 22 symptom domains at baseline (black bars) and after approximately 3–4 months of nVNS therapy (red bars). Each domain is rated on a 0–4 scale, with higher scores indicating greater symptom severity. * *Wilcoxon signed-rank test, q*<0.05 *(FDR- corrected)*. **(B)** Average change in NSI item scores, calculated as follow-up minus baseline for each individual. Negative values reflect improvement. **(C)** NSI symptoms are grouped into four broader domains (Affective, Cognitive, Somatic, Vestibular). Bars show total magnitude change in broader domains averaged across patients. Patients with mTBI + PTSD exhibited the largest mean reductions within the Affective and Cognitive domains compared to patients without suspected PTSD, indicating preferential improvement in emotional and cognitive symptom clusters for those with PTSD. Values represent mean ± standard error of the mean (SEM).

Among patients with comorbid mTBI + PTSD, adjunctive nVNS use was associated with statistically significant reductions in NSI scores over 3–4 months of treatment. Mean total NSI scores decreased from 2.50 ± 0.60 at baseline to 2.03 ± 0.46 at follow-up (Table 1; false discovery rate [FDR]-corrected). Out of 22 symptoms, 13 were significantly reduced after correcting from multiple comparisons (Figures 1A,B). Reductions were most pronounced within affective and cognitive domains (Figure 1C), where mean item scores decreased by −0.58 (*p* = 0.010, FDR-corrected) and −0.64 (*p* = 0.0152, FDR-corrected), respectively. The largest individual item change was observed for *feeling easily overwhelmed* (−0.71; *p* = 0.0173, FDR-corrected). Somatic (−0.34; *p* = 0.0396, FDR-corrected) and vestibular (−0.58; *p* = 0.0495, FDR-corrected) domains were also significantly reduced although to a lesser degree.

Consistent with prior findings, neither age at injury nor variability in NSI follow-up timing was associated with baseline severity or magnitude of symptom change (data not shown; ANOVA).

### 3.3 Responder analysis across NSI symptom domains

**Figure 2:**
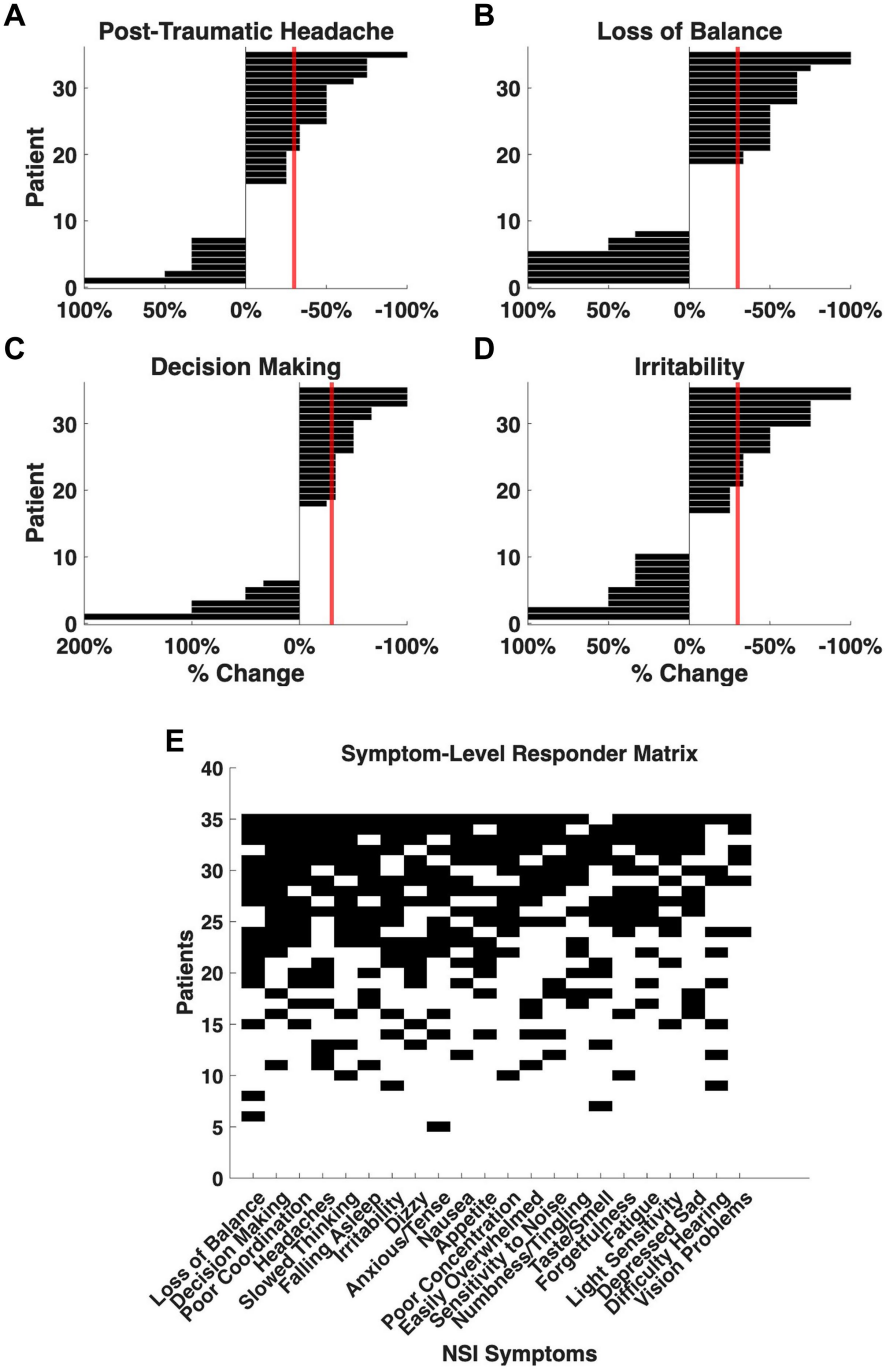
Clinically meaningful improvement in symptom severity following adjunctive nVNS in patients with mTBI + PTSD. Individual changes in NSI scores were evaluated to determine treatment response. Symptom improvement was expressed as the percentage change from baseline, and participants achieving a ≥30% reduction in severity (crossing the red threshold line) were classified as responders. **(A)** Post-traumatic headache: 43% of patients (*n* = 15) met responder criteria. **(B)** Loss of balance: 49% (*n* = 17). **(C)** Decision making: 49% (*n* = 17). **(D)** Irritability: 43% (*n* = 15). **(E)** Binary response matrix depicting responder status across all symptoms and participants. Rows correspond to individual patients (*n* = 35 for the mTBI + PTSD subgroup), and columns represent NSI items. Black squares denote symptom-specific responses, with the total number of responses ordered top (high) to bottom (low) and number of symptoms that met responder criteria ordered left (high) to right (low).

Responder analysis, defined as a ≥30% reduction in individual symptom severity, identified a subset of participants meeting criteria for clinically meaningful change across multiple NSI domains (Figure 2). A reduction of 30% or more is widely accepted as a threshold for meaningful improvement across a variety of conditions including psychiatric (Leon et al., 2001), chronic pain (Dworkin et al., 2008), neurodegenerative (Baudendistel and Earhart, 2025), and importantly subjective clinical questionnaires like the NSI (Troy et al., 2023).

Forty percent of patients with mTBI + PTSD met the ≥30% total NSI reduction criterion, comparable to response rates observed in the full mTBI cohort. The highest responder frequencies were observed for *loss of balance* (49%), *decision making* (49%), and *poor coordination* (46%). Additionally, 43% of patients met responder criteria for *post-traumatic headaches, slowed thinking, difficulty falling asleep* and *irritability*. An additional sensitivity analysis was run using ≥40%, ≥50%, and ≥60% thresholds (Supplementary Table 4). Patterns of symptom response overlapped substantially with those previously reported, though affective and cognitive items demonstrated a greater relative contribution to total improvement in the PTSD subgroup (Figure 1C).

Twenty-nine percent of mTBI + PTSD patients met responder criteria in 11 or more symptoms and 69% met responder criteria in at least 1 symptom. These rates are lower than those reported for the full cohort (34 and 90% respectively, Ament et al., 2025) and which may reflect differences in baseline symptom burden. We removed the mTBI + PTSD from the full mTBI cohort and found that responder rates (33 and 91%) were not significantly altered in the remaining patients (*n* = 64).

Normative values for pooled NSI domains have been reported for mTBI populations (Soble et al., 2014). In the mTBI + PTSD cohort, 6% of patients moved from an “elevated symptom” category at baseline to “clinically borderline” after treatment for the vestibular and somatic domains. Twenty-six percent of patients improved to “clinically borderline” in the affective domain and 31% in the cognitive domain.

### 3.4 Baseline symptom severity predicts treatment-related change

**Figure 3:**
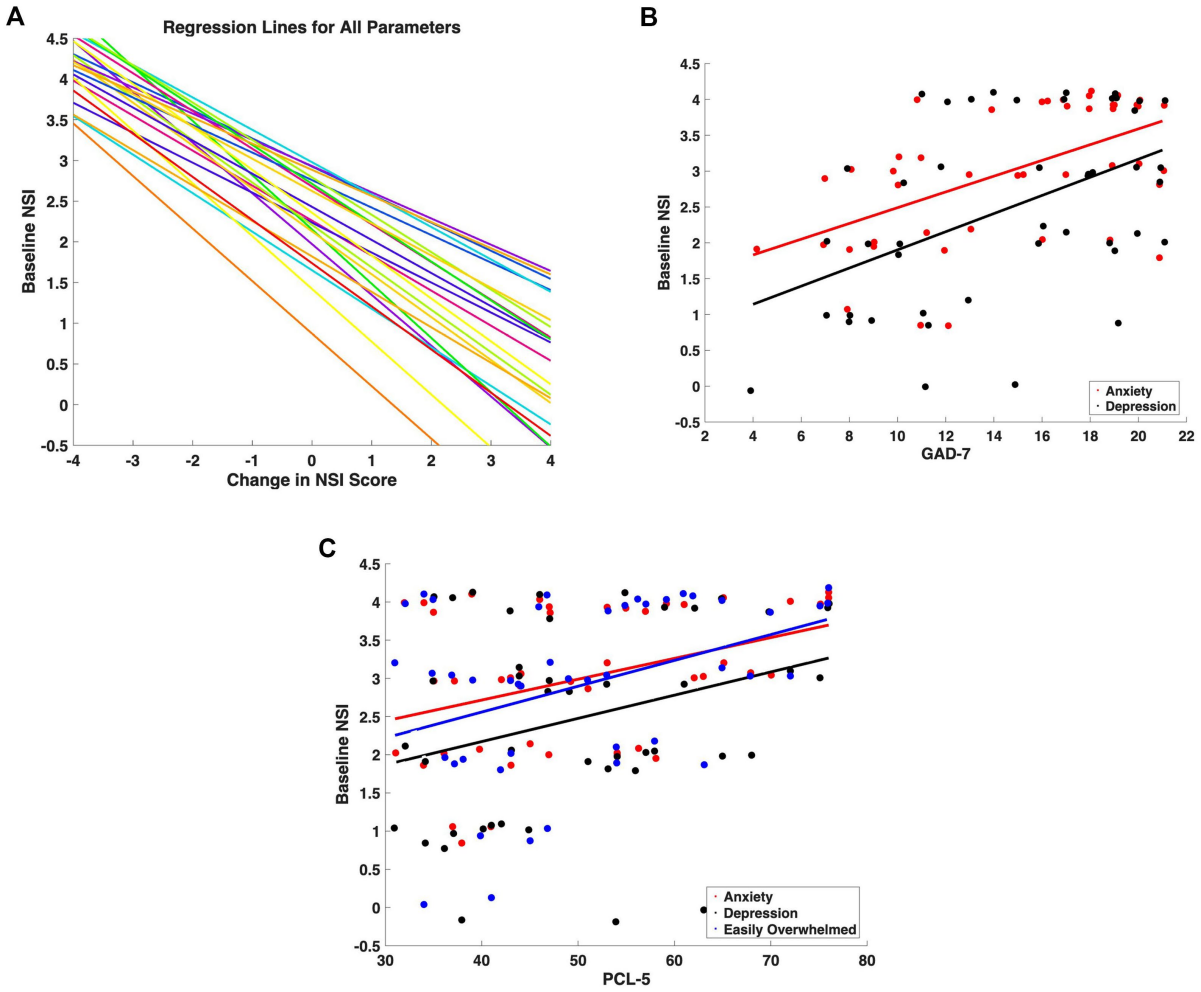
Associations between baseline NSI scores and clinical measures of anxiety and post-traumatic stress in patients with mTBI + PTSD. **(A)** Regression lines showing the correlation between initial severity and magnitude of change for each symptom. **(B)** Correlations between baseline NSI scores for *anxiety* (red) and *depression/sadness* (black) and total scores on the GAD-7. **(C)** Correlations between baseline NSI scores for anxiety (red), depression/sadness (black), and *feeling easily overwhelmed* (blue) and total scores on the PCL-5. Linear regression lines are shown in corresponding colors.

Baseline symptom severity correlated inversely with treatment-related change, indicating that higher baseline severity was associated with larger absolute changes in symptom scores (Figure 3A). The average Pearson’s correlation across all 22 symptoms was −0.57 (range: −0.41 to −0.73).

To account for potential regression-to-the-mean effects, baseline-adjusted ANCOVA models were performed. Baseline NSI significantly predicted follow-up NSI (*β* = 0.76, *p* < 0.001). However, no significant differences in follow-up severity were observed between mTBI-only and mTBI + PTSD groups after adjustment (*p* = 0.74), and Baseline × Group interactions were not significant (*p* = 0.20), supporting homogeneity of regression slopes across cohorts.

### 3.5 Baseline affective symptoms correlate with anxiety and PTSD measures

Given the heavier symptom burden in patients with mTBI and comorbid PTSD, we correlated baseline NSI scores for *anxiety* and *depression/sadness* with GAD-7 totals (*r* = 0.55 and 0.48, respectively; Figure 3B). Similarly, baseline *anxiety*, *depression/sadness*, and *feeling easily overwhelmed* were each positively associated with PCL-5 scores (*r* = 0.31–0.39; Figure 3C).

### 3.6 Principal component and clustering analyses reveal distinct symptom profiles and PTSD distribution

**Figure 4:**
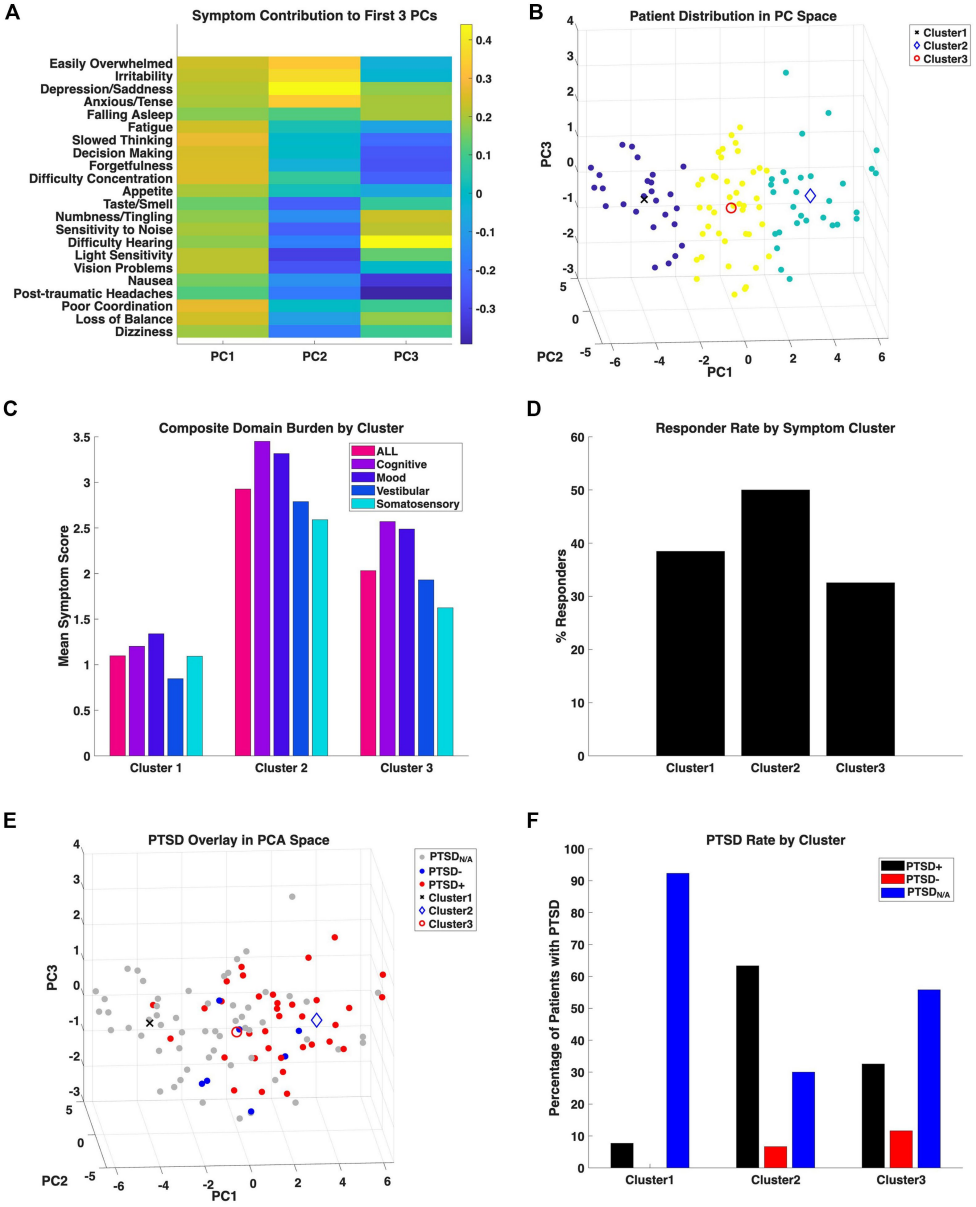
Principal component analysis of symptom structure across the mTBI cohort. **(A)** Heat map illustrating the relative weighting of each NSI item across the first three principal components (PC1–PC3) derived from all patients (*n* = 102). Color intensity represents the direction and magnitude of each variable’s loading on the component axes. PC1 predominantly represented global symptom burden with stronger loadings from affective and cognitive items, PC2 captured variability in affective vs. somatosensory + vestibular symptoms, and PC3 differentiated residual somatic-cognitive vs. sensory-emotional variance. **(B)** Three-dimensional PCA score plot showing unsupervised clustering of participants using *k*-means (*k* = 3). Each point represents an individual patient, colored by cluster assignment. The three clusters corresponded to distinct symptom profiles, differentiating patients with relatively mild, mixed, and high symptom loads. **(C)** Bar graphs show the average symptom severity separated by symptom domain and PCA clustering. **(D)** Bars show the percentage of patients that met responder criteria for *total score* within each cluster. **(E)** PCA score plot showing the distribution of patients with comorbid PTSD (red) relative to those who did not meet PTSD criteria (blue) or were missing PCL-5 data (gray). The overlay demonstrates that patients meeting PTSD criteria are concentrated on the positive axis of PC1 and indicates greater baseline symptom burden within this subgroup. **(F)** Proportion of patients meeting PTSD criteria (black) within each cluster. The highest PTSD prevalence occurred in Cluster 2 and was characterized by elevated symptom load, consistent with the spatial distribution observed in **E**.

Principal component analysis (PCA) of baseline NSI scores from all 102 participants identified three primary components explaining 56% of total variance (Figure 4A). The first principal component (PC1) accounted for 42.4% of variance and reflected global symptom severity. PC2 (8.2%) captured affective versus somatosensory variability, while PC3 (5.9%) represented residual cognitive–somatic versus affective–sensory differentiation.

K-means clustering (*k* = 3) applied to PCA scores delineated three subgroups corresponding to mild, mixed, and high symptom burden profiles (Figure 4B). Cluster 2 demonstrated the greatest overall symptom severity, plotting furthest along the positive axis of PC1 (Figures 4B,C). This cluster also exhibited the highest frequency of clinical responders and the largest absolute symptom change following intervention (Figure 4D).

Bootstrap resampling (2,000 iterations) demonstrated high stability of PC1, with narrow 95% confidence intervals for loadings (mean width 0.076; range 0.039–0.126) and consistent loading patterns across resamples. In contrast, PC2 and PC3 explained substantially less variance and demonstrated greater variability in bootstrap loadings, indicating weaker and more sample-dependent secondary structure.

Overlay of PTSD status revealed that patients meeting PCL-5 criteria were concentrated along the positive end of PC1 and predominantly mapped to Cluster 2 (Figures 4E,F). The prevalence of probable PTSD was 63% in Cluster 2, compared with 33% in the mixed cluster and 8% in the mild cluster. Approximately 30% of patients within Cluster 2 were missing PCL-5 data; however, only a small minority (<7%) of Cluster 2 patients with available data did not meet PTSD criteria, suggesting a strong association between elevated symptom burden and probable PTSD.

Overall, separation by PTSD status was primarily aligned with PC1, supporting the interpretation that PTSD is associated with increased global symptom burden rather than a distinct orthogonal symptom subtype.

## 4 Discussion

In this retrospective observational analysis, adjunctive nVNS was associated with symptom improvement that met predefined thresholds for clinical meaningfulness in patients with chronic mTBI who met screening criteria for probable PTSD, a subgroup characterized by substantially greater baseline symptom burden than the broader mTBI population. Over 3–4 months of treatment, improvements were observed across affective, cognitive, somatic, and vestibular domains, with affective and cognitive symptoms accounting for a disproportionate share of total improvement. Nearly one-third of patients experienced broad multisymptom benefit, and improvement magnitude scaled strongly with baseline symptom severity, indicating that patients with the greatest clinical burden demonstrated comparable absolute symptom reductions. Notably in PCA results, individuals with probable PTSD were concentrated among those with the highest global symptom load yet demonstrated robust absolute reductions in symptom severity. This suggests that the heavier symptom burden associated with PTSD does not preclude therapeutic responsiveness. Together, these findings refine prior observations in heterogeneous mTBI cohorts by demonstrating that adjunctive nVNS may be particularly relevant for patients with overlapping neurological and psychiatric symptom dysregulation—an often refractory population in which targeted neuromodulation may address shared underlying pathophysiology.

### 4.1 Multivariate symptom structure and PTSD-enriched phenotypes

Multivariate analyses (i.e., PCA) further contextualize these findings by revealing distinct symptom profiles within the broader mTBI cohort. PCA of baseline NSI scores identified a dominant axis reflecting global symptom burden, with secondary components capturing affective versus somatosensory and cognitive–sensory variability. Unsupervised clustering based on these components delineated three symptom profiles ranging from relatively mild to heavily symptom burdened. Patients meeting screening criteria for probable PTSD were concentrated within the highest-burden cluster, which was also characterized by the greatest proportion of clinical responders. This convergence of high baseline severity (NSI at intake), PTSD enrichment (PCL-5 ≥ 31), and treatment responsiveness (change in NSI) suggests that PCL-5 scores may be less important as an isolated diagnosis than as a marker of a broader, high-burden symptom phenotype that remains modifiable through targeted intervention.

It is important to note that several NSI affective items (e.g., anxiety, irritability, depressed mood) and select cognitive items (e.g., concentration difficulty) overlap conceptually with PTSD symptom domains. As such, observed improvements within affective and certain cognitive domains may reflect changes in shared symptom constructs rather than discrete diagnostic categories. Interpretation of these domain-level findings should therefore consider the overlapping symptom architecture between persistent mTBI and PTSD.

Although PTSD screening data were unavailable for a subset of patients within the highest-burden cluster, the low prevalence of non-PTSD cases among those with complete data suggests that PTSD-related symptomatology may be underrecognized in this group. Nonetheless, the incomplete availability of PCL-5 data represents an important limitation and precludes definitive conclusions regarding PTSD prevalence across all clusters.

These clustering patterns describe baseline symptom structure and associated response trajectories but should not be interpreted as predictive or treatment-defining without prospective validation. Therefore, these findings should be interpreted as hypothesis-generating and underscore the need for more systematic psychiatric characterization in future studies.

### 4.2 Mechanistic considerations: vagal modulation of shared mTBI–PTSD circuitry

The pattern of symptom change is directionally consistent with hypotheses regarding neurobiological mechanisms through which vagus nerve stimulation may influence both mTBI- and PTSD-related pathology. The vagus nerve provides a critical interface between peripheral autonomic signaling and central networks involved in emotion regulation, stress responsivity, and cognitive control (Staats and Blake, 2024). Afferent vagal projections to brainstem nuclei such as the nucleus tractus solitarius and locus coeruleus modulate noradrenergic tone, limbic excitability, and cortical network dynamics, all of which are implicated in the persistence of post-concussive and post-traumatic stress symptoms (Bremner, 2007; Dieter and Engel, 2019; Daugherty et al., 2024). In addition, vagal activation engages the cholinergic anti-inflammatory pathway, suppressing pro-inflammatory cytokine release in preclinical and translational models (Lerman et al., 2016; Tornero et al., 2022) and potentially mitigating neuroinflammatory priming that has been linked to both chronic TBI symptoms and PTSD-related cognitive and affective dysfunction.

Within this framework, the preferential improvement of affective and cognitive symptoms in the PTSD-enriched subgroup is notable. Emotional dysregulation, heightened stress reactivity, and attentional impairment are core features of PTSD and are strongly influenced by autonomic and limbic circuitry (Daugherty et al., 2024). While the present study does not directly assess physiological markers of autonomic or inflammatory modulation, the observed symptom trajectories are directionally consistent with the hypothesis that nVNS may help normalize dysregulated stress-response networks in patients with overlapping neurological and psychiatric vulnerability. Importantly, these mechanistic considerations remain speculative and should be tested explicitly in future prospective studies incorporating physiological and biomarker endpoints.

A final consideration is the overlap among symptom domains in mTBI and PTSD and the possibility that shared mechanistic dysfunction may produce additive symptom burden. In the present mTBI + PTSD cohort, 43% of patients reported clinically meaningful improvement in difficulty *falling asleep*. Sleep disturbance is common following mTBI and is associated with greater somatic, cognitive, and affective symptom severity, as well as prolonged recovery trajectories. Emerging work also suggests that disrupted sleep after mTBI may impair glymphatic clearance and contribute to persistent neurocognitive symptoms (Piantino et al., 2022). Although sleep outcomes were not a primary focus of the present analysis, the observed improvement in sleep initiation may be relevant given the reciprocal relationships among sleep regulation, emotional processing, and cognitive function. These interrelated pathways further support the rationale for therapeutic approaches that modulate shared physiological systems—such as nVNS—rather than targeting individual symptoms in isolation.

### 4.3 Strengths and limitations

Several strengths of this study warrant consideration. The use of naturalistic clinical data enhances ecological validity and reflects real-world treatment conditions in a population with persistent, clinically significant symptoms. Symptom assessment was conducted using a validated, multidimensional instrument, and analyses incorporated correction for multiple comparisons. The integration of both univariate and multivariate approaches enabled a more nuanced characterization of symptom structure and treatment response beyond total score changes alone. The broad symptom interrogation by the NSI also provides critical information on symptoms which are not commonly studied. For example, the inclusion of vestibular symptoms like *balance* and *coordination* provides information on symptoms that are not typically tracked in neuromodulation studies.

Important limitations must also be acknowledged. The retrospective observational design precludes causal inference, and the absence of a sham-controlled comparator introduces the possibility of placebo effects or nonspecific treatment influences. Standard-of-care interventions were individualized and not standardized across patients, creating potential confounding from concurrent therapies. PTSD classification was based on a selectively administered screening instrument, resulting in incomplete ascertainment and potential selection bias. Subgroup analyses were conducted post-hoc and should therefore be interpreted as exploratory. Moreover, longitudinal changes in PTSD-specific symptom measures were not assessed; therefore, findings should not be interpreted as evidence of PTSD symptom remission or diagnostic resolution.

Because enrollment favored patients with elevated baseline symptom burden, regression to the mean may have contributed to overall symptom reductions. However, baseline-adjusted ANCOVA models demonstrated strong baseline–follow-up associations with parallel slopes and no differential group effects, suggesting that improvements were not attributable to phenotype-specific statistical regression. Nonetheless, randomized controlled trials are necessary to confirm treatment-specific effects and to fully disentangle biological response from statistical and expectancy-related influences.

### 4.4 Future directions and conclusions

Despite these limitations, the current analysis provides clinically relevant insights into a population frequently excluded from controlled trials and underserved by existing treatment paradigms. The findings indicate that adjunctive nVNS was well tolerated and associated with symptom reductions in patients with chronic mTBI and comorbid PTSD. Notably, individuals with higher baseline symptom burden demonstrated comparable absolute improvements, suggesting that elevated initial severity did not preclude observed symptom change in this cohort.

Future prospective, controlled studies are needed to validate these observations and clarify the role of nVNS in trauma-related neuropsychiatric rehabilitation. Such studies should incorporate systematic PTSD assessment, repeated longitudinal measurement of psychiatric and cognitive outcomes, and objective biomarkers of autonomic and inflammatory modulation. Evaluation of functional endpoints, including sleep quality, return to work, and quality of life, will be essential to determine the broader clinical impact and durability of benefit. Together, these efforts will help define whether vagal neuromodulation can play a targeted role in addressing the shared pathophysiology underlying persistent mTBI symptoms and post-traumatic stress.

## Data Availability

The raw data supporting the conclusions of this article will be made available by the authors, without undue reservation.
