# Supplementary material for: Retrospective post-hoc subgroup analysis of adjunctive non-invasive vagus nerve stimulation in chronic mTBI with comorbid PTSD
Source: Front Neurosci. 2026 Apr 13;20:1808542. doi: 10.3389/fnins.2026.1808542 (PMC13111240; doi:10.3389/fnins.2026.1808542)
Supplement: Supplementary file 1 [file Table_1.docx]

| **Patient Number** | **PCL-5 Score** | **Vestibular Score 1** | **Vestibular Score 2** | **Somatic Score 1** | **Somatic Score 2** | **Cognitive Score 1** | **Cognitive Score 2** | **Affective Score 1** | **Affective Score 2** | **Total Score1** | **Total Score2** |
| --- | --- | --- | --- | --- | --- | --- | --- | --- | --- | --- | --- |
| **1** | 54 | 11 | 5 | 23 | 11 | 13 | 6 | 12 | 10 | 62 | 35 |
| **2** | 53 | 5 | 9 | 10 | 17 | 10 | 10 | 15 | 21 | 41 | 59 |
| **3** | 46 | 5 | 4 | 6 | 5 | 12 | 8 | 24 | 11 | 49 | 28 |
| **4** | 51 | 7 | 4 | 19 | 6 | 13 | 11 | 18 | 13 | 61 | 37 |
| **5** | 65 | 6 | 9 | 16 | 16 | 12 | 12 | 17 | 17 | 55 | 57 |
| **6** | 70 | 10 | 12 | 23 | 23 | 14 | 15 | 21 | 24 | 71 | 78 |
| **7** | 44 | 6 | 3 | 19 | 8 | 13 | 4 | 16 | 8 | 58 | 25 |
| **8** | 42 | 5 | 11 | 15 | 10 | 16 | 7 | 16 | 3 | 55 | 33 |
| **9** | 34 | 5 | 4 | 13 | 9 | 14 | 8 | 16 | 11 | 51 | 34 |
| **10** | 72 | 9 | 9 | 15 | 22 | 10 | 13 | 16 | 23 | 55 | 72 |
| **11** | 31 | 4 | 8 | 16 | 14 | 10 | 11 | 13 | 11 | 45 | 47 |
| **12** | 37 | 1 | 4 | 9 | 10 | 8 | 8 | 10 | 6 | 29 | 28 |
| **13** | 43 | 10 | 8 | 12 | 20 | 12 | 16 | 18 | 21 | 54 | 71 |
| **14** | 75 | 10 | 8 | 22 | 12 | 10 | 6 | 22 | 9 | 70 | 40 |
| **15** | 34 | 9 | 1 | 15 | 12 | 7 | 5 | 13 | 8 | 46 | 27 |
| **16** | 55 | 10 | 6 | 17 | 12 | 16 | 5 | 23 | 16 | 69 | 41 |
| **17** | 47 | 5 | 1 | 11 | 10 | 10 | 5 | 14 | 8 | 42 | 27 |
| **18** | 43 | 2 | 8 | 4 | 7 | 7 | 4 | 11 | 3 | 25 | 25 |
| **19** | 59 | 10 | 5 | 14 | 10 | 12 | 3 | 23 | 12 | 63 | 36 |
| **20** | 76 | 9 | 9 | 22 | 20 | 16 | 12 | 24 | 18 | 73 | 63 |
| **21** | 37 | 4 | 4 | 4 | 4 | 9 | 9 | 19 | 12 | 38 | 31 |
| **22** | 38 | 4 | 5 | 13 | 13 | 9 | 14 | 12 | 17 | 42 | 54 |
| **23** | 61 | 5 | 5 | 15 | 16 | 10 | 10 | 21 | 21 | 55 | 56 |
| **24** | 47 | 12 | 2 | 20 | 9 | 16 | 2 | 24 | 1 | 80 | 16 |
| **25** | 58 | 9 | 6 | 15 | 18 | 8 | 14 | 15 | 22 | 50 | 65 |
| **26** | 44 | 8 | 3 | 8 | 5 | 9 | 9 | 18 | 17 | 46 | 36 |
| **27** | 62 | 8 | 4 | 11 | 11 | 14 | 10 | 19 | 13 | 58 | 39 |
| **28** | 68 | 9 | 10 | 11 | 15 | 11 | 16 | 17 | 21 | 49 | 63 |
| **29** | 39 | 9 | 9 | 19 | 14 | 11 | 12 | 21 | 18 | 66 | 57 |
| **30** | 32 | 8 | 2 | 11 | 1 | 15 | 2 | 21 | 3 | 55 | 8 |
| **31** | 53 | 6 | 6 | 20 | 16 | 15 | 11 | 23 | 16 | 69 | 53 |
| **32** | 54 | 8 | 7 | 16 | 15 | 11 | 11 | 12 | 13 | 51 | 51 |
| **33** | 35 | 7 | 7 | 14 | 19 | 9 | 6 | 20 | 21 | 54 | 59 |
| **34** | 56 | 8 | 4 | 16 | 13 | 15 | 12 | 16 | 14 | 59 | 46 |
| **35** | 65 | 12 | 7 | 24 | 17 | 15 | 14 | 24 | 21 | 81 | 64 |

**Supplemental Table 1: Patient Scores**

PCL-5 and composite NSI domains at baseline (1) and follow-up (2) are shown for each patient.
