# Supplementary material for: Retrospective post-hoc subgroup analysis of adjunctive non-invasive vagus nerve stimulation in chronic mTBI with comorbid PTSD
Source: Front Neurosci. 2026 Apr 13;20:1808542. doi: 10.3389/fnins.2026.1808542 (PMC13111240; doi:10.3389/fnins.2026.1808542)
Supplement: Supplementary file 2 [file Table_2.docx]

| **Patient Number** | **Psychotherapy exposure** | **Psychiatric Medications** | **Vestibular Therapy** | **Vision Therapy** |
| --- | --- | --- | --- | --- |
| **1** | None | None | Yes | Yes |
| **2** | Prior | Prior | No | Yes |
| **3** | None | None | No | Yes |
| **4** | None | None | Yes | No |
| **5** | Concurrent | Concurrent | Yes | Yes |
| **6** | Concurrent | Concurrent | Yes | Yes |
| **7** | Prior | Concurrent | Yes | Yes |
| **8** | None | None | No | Yes |
| **9** | None | None | No | Yes |
| **10** | Prior | Concurrent | Yes | No |
| **11** | Concurrent | None | No | No |
| **12** | Concurrent | None | No | Yes |
| **13** | Concurrent | Concurrent | No | Yes |
| **14** | Concurrent | Concurrent | Yes | Yes |
| **15** | None | Concurrent | No | No |
| **16** | Concurrent | Concurrent | Yes | Yes |
| **17** | None | None | Yes | Yes |
| **18** | Concurrent | Concurrent | No | Yes |
| **19** | Concurrent | Concurrent | Yes | No |
| **20** | Prior | Concurrent | No | No |
| **21** | Concurrent | None | No | Yes |
| **22** | Concurrent | Concurrent | No | Yes |
| **23** | Concurrent | Concurrent | No | No |
| **24** | None | Concurrent | No | Yes |
| **25** | None | None | Yes | No |
| **26** | Prior | Concurrent | No | No |
| **27** | Prior | Concurrent | Yes | Yes |
| **28** | Prior | Concurrent | Yes | Yes |
| **29** | Concurrent | None | No | No |
| **30** | None | Concurrent | No | Yes |
| **31** | Concurrent | None | Yes | Yes |
| **32** | Prior | Concurrent | Yes | Yes |
| **33** | Concurrent | Concurrent | No | Yes |
| **34** | Concurrent | Concurrent | No | Yes |
| **35** | None | None | No | Yes |

**Supplemental Table 2: Individual patient exposure to other therapies**

Each patient’s exposure to psychotherapy and psychiatric medications was categorized as ‘None’, ‘Prior’, or ‘Concurrent’. Additional administration of therapies for vestibular or vision symptoms are also reported.
