# Supplementary material for: Retrospective post-hoc subgroup analysis of adjunctive non-invasive vagus nerve stimulation in chronic mTBI with comorbid PTSD
Source: Front Neurosci. 2026 Apr 13;20:1808542. doi: 10.3389/fnins.2026.1808542 (PMC13111240; doi:10.3389/fnins.2026.1808542)
Supplement: Supplementary file 3 [file Table_3.docx]

| **Vestibular Score** | Dizziness |
| --- | --- |
|  | Loss of Balance |
|  | Poor Coordination |
| **Somatic Score** | Post-Traumatic Headaches |
|  | Nausea |
|  | Vision Problems |
|  | Light Sensitivity |
|  | Sensitivity to Noise |
|  | Numbness/Tingling |
|  | Altered Taste/Smell |
| **Cognitive Score** | Poor Concentration |
|  | Forgetfulness |
|  | Decision Making |
|  | Slowed Thinking |
| **Affective Score** | Fatigue |
|  | Falling Asleep |
|  | Anxious/Tense |
|  | Depressed Sad |
|  | Irritability |
|  | Easily Overwhelmed |
| **Uncategorized** | Difficulty Hearing |
|  | Appetite |

**Supplemental Table 3: Individual NSI Symptoms are grouped into composite domains**
