# Supplementary material for: Retrospective post-hoc subgroup analysis of adjunctive non-invasive vagus nerve stimulation in chronic mTBI with comorbid PTSD
Source: Front Neurosci. 2026 Apr 13;20:1808542. doi: 10.3389/fnins.2026.1808542 (PMC13111240; doi:10.3389/fnins.2026.1808542)
Supplement: Supplementary file 4 [file Table_4.docx]

| **NSI Domains** | ≥**30% Reduction** | ≥**40% Reduction** | ≥**50% Reduction** | ≥**60% Reduction** |
| --- | --- | --- | --- | --- |
| **Dizziness** | 40 | 31 | 31 | 11 |
| **Loss of Balance** | 49 | 43 | 43 | 23 |
| **Poor Coordination** | 46 | 34 | 34 | 20 |
| **Post-Traumatic Headaches** | 43 | 31 | 31 | 14 |
| **Nausea** | 37 | 34 | 34 | 23 |
| **Vision Problems** | 17 | 14 | 14 | 9 |
| **Light Sensitivity** | 31 | 20 | 20 | 11 |
| **Difficulty Hearing** | 26 | 23 | 23 | 9 |
| **Sensitivity to Noise** | 34 | 26 | 26 | 17 |
| **Numbness/Tingling** | 34 | 29 | 29 | 26 |
| **Altered Taste/Smell** | 34 | 31 | 31 | 17 |
| **Appetite** | 37 | 29 | 29 | 14 |
| **Poor Concentration** | 37 | 29 | 29 | 23 |
| **Forgetfulness** | 34 | 29 | 29 | 14 |
| **Decision Making** | 49 | 29 | 29 | 14 |
| **Slowed Thinking** | 43 | 29 | 29 | 11 |
| **Fatigue** | 34 | 14 | 14 | 11 |
| **Falling Asleep** | 43 | 29 | 29 | 9 |
| **Anxious/Tense** | 40 | 29 | 29 | 6 |
| **Depressed Sad** | 31 | 26 | 26 | 11 |
| **Irritability** | 43 | 29 | 29 | 17 |
| **Easily Overwhelmed** | 37 | 31 | 31 | 14 |
| **Vestibular Score** | 40 | 37 | 29 | 14 |
| **Somatic Score** | 26 | 17 | 14 | 6 |
| **Cognitive Score** | 37 | 31 | 23 | 14 |
| **Affective Score** | 46 | 29 | 20 | 11 |
| **Total Score** | 41 | 29 | 9 | 6 |

**Supplemental Table 4: Sensitivity Analysis for Responder Criteria**

Additional analysis using various thresholds for meaningful improvement were undertaken for each individual and composite NSI symptom.
